# O-26 - IMPROVING SUPPORT FOR SEX WORKERS IN WALSALL RESEARCH PROJECT. INSIGHTS FROM LIVED EXPERIENCE AND LOCAL SERVICE PROVIDERS AND COMMISSIONERS

**DOI:** 10.1093/pubmed/fdag046.023

**Published:** 2026-07-09

**Authors:** Mr Nigel Smith, Ms Danielle Jayes

**Affiliations:** UK Health Security Agency West Midlands; UK Health Security Agency West Midlands

## Abstract

**Background:**

Sex workers in Walsall experience significant and intersecting health inequalities shaped by stigma, trauma, unstable housing, policing practices, fragmented services, and inconsistent access to mental health support.

**Objectives:**

This study was coproduced by UKHSA West Midlands, Walsall Council, and Changing Lives. With the objective demonstrating how individual, interpersonal, community and structural factors combine to influence access to care and how these results can be used to influence service planning.

**Methods:**

Interviews with women engaged in sex work; surveys, focus groups and interviews with local commissioners and providers.

**Results:**

Insights from women, local providers/commissioners show that the current system is often difficult to navigate, inflexible, and not trauma informed, leading many women to disengage from support. Women described stigma in healthcare, complex housing pathways, mixed experiences with police, and limited mental health support.

Providers highlighted short term funding, unclear referral pathways, and reliance on the voluntary sector to fill gaps. Across all groups, consistent outreach, trusting relationships, and women only safe spaces were identified as the strongest enablers of engagement.

**Conclusions:**

A more coherent, trauma informed and person-centred system is required. The insights from this study provide a strong evidence base for shaping future commissioning decisions across public sector services.

The findings highlight the importance of commissioning for consistency, with stable, long-term funding that protects outreach, drop-ins, and specialist support. They also point to the need for co-located and flexible access models, including evening outreach, women-only hubs and informal safe spaces that reduce barriers created by rigid appointment systems.

Embedding lived experience and frontline insight within commissioning cycles and strategies is essential to ensure services reflect women’s realities and provides support for a more equitable/responsive system that supports the health, safety and wellbeing of sex workers.

